# Efficacy of *ω*-3 Polyunsaturated Fatty Acids in Patients with Lung Cancer Undergoing Radiotherapy and Chemotherapy: A Meta-Analysis

**DOI:** 10.1155/2022/6564466

**Published:** 2022-07-08

**Authors:** Xin Tao, Qiang Zhou, Zhiyong Rao

**Affiliations:** ^1^Department of Clinical Nutrition, Suining Central Hospital, Suining, China; ^2^Department of Oncology, Suining Central Hospital, Suining, China; ^3^Department of Clinical Nutrition, West China Hospital of Sichuan University, Chengdu, China

## Abstract

**Background:**

Radiotherapy and chemotherapy in patients with lung cancer can lead to a series of problems such as malnutrition and inflammatory reaction. Some studies have shown that *ω*-3 polyunsaturated fatty acids (PUFAs) could improve malnutrition and regulate inflammatory reaction in these patients, but no relevant meta-analysis exists.

**Methods:**

We systematically searched randomized controlled trials of *ω*-3 PUFAs in the adjuvant treatment of lung cancer in the PubMed, EMBASE, Cochrane Library, Web of Science, Chinese Biomedical Literature Database (CBM), China National Knowledge Infrastructure (CNKI), and Wanfang databases. Relevant outcomes were extracted, and we pooled standardized mean differences (SMDs) using a random or fixed-effects model. The risk of bias was evaluated according to the Cochrane Handbook (version 15.1). The quality of evidence was assessed using the Grading of Recommendations Assessment, Development, and Evaluation (GRADE).

**Results:**

A total of 7 studies were included. The SMDs (95% CI) of body weight change, albumin change, energy intake, and protein intake at the end of intervention were 1.15 (0.50, 1.80), 0.60 (0.11, 1.09), 0.39 (−0.10, 0.89), and 0.27 (−0.04, 0.58), respectively. The SMDs (95% CI) of CRP change and TNF-*α* change were −3.44 (−6.15, −0.73) and −1.63 (−2.53, −0.73), respectively.

**Conclusions:**

*ω*-3 PUFAs can improve nutritional status and regulate indicators of inflammation in patients with lung cancer undergoing radiotherapy and chemotherapy. This study was registered in the PROSPERO (registration number: CRD42022307699).

## 1. Introduction

Lung cancer is one of the most common malignant tumors, which is difficult to treat, and has high mortality. In 2020, 2.21 million lung cancer patients were newly diagnosed worldwide and 1.8 million patients died of lung cancer. Lung cancer has become the leading cause of cancer death in mankind [1]. Radiotherapy and chemotherapy are the most common treatments for lung cancer. However, these treatments may alter patients' nutritional status and inflammatory state, affecting their quality of life and prognosis. Cancer patients with malnutrition have higher complication rates, longer length of stay, and worse clinical outcome [2]. Cancer patients with high levels of inflammation have lower overall survival, disease-free survival, and progression-free survival [3].

*ω*-3 PUFAs are essential fatty acids that the human body cannot synthesize and are abundant in vegetable oils and fish fats. Docosahexaenoic acid (DHA) and eicosapentaenoic acid (EPA) are important components of *ω*-3 PUFAs, and they are also the most studied *ω*-3 PUFAs. *ω*-3 PUFAs have a certain role in preventing cardiovascular disease, adjusting inflammation, and improving nutritional status [4]. Mocellin et al.'s study showed that *ω*-3 PUFAs increased plasma albumin and prealbumin levels in gastric cancer patients [5]. Ma's research showed that *ω*-3 PUFAs downregulated CRP levels and reduced the duration of the systemic inflammatory response syndrome (SIRS) [6]. A meta-analysis showed that *ω*-3 PUFAs could improve the nutritional status of patients after gastrectomy of gastric cancer and downregulate the levels of inflammatory indicators, such as C-reactive protein (CRP) and interleukin 6 (IL-6) [7]. However, studies by Lam CN and Carvalho TC showed that *ω*-3 PUFAs had no significant effect on nutritional improvement and inflammation regulation in cancer patients [8, 9]. Some randomized controlled trials have studied the therapeutic effect of *ω*-3 PUFAs on patients with lung cancer during radiotherapy and chemotherapy, but there is no corresponding meta-analysis study. This meta-analysis explores the efficacy of *ω*-3 PUFAs in patients treated with lung cancer undergoing radiotherapy and chemotherapy, so as to provide a reference for the treatment of lung cancer patients.

## 2. Methods

### 2.1. Search Strategy

Our research follows Preferred Reporting Items for Systematic Reviews and Meta-Analyses (PRISMA). We searched the PubMed, Embase, Cochrane Library, Web of Science, CBM, CBM, CNKI, and Wanfang databases. The retrieval method is a combination of subject words and free words. The search keywords were “omega-3 polyunsaturated fatty acids, *ω*-3 PUFAs, fish oil, docosahexaenoic acid, DHA, eicosapentaenoic acid, EPA, lung cancer, lung tumor, lung Neoplasms, lung carcinoma, radiotherapy, chemotherapy, chemoradiotherapy.” No language limits were involved. The retrieval time is from the establishment of each database to February 2022. The search strategy can be found in Supplementary Table 2.

### 2.2. Literature Selection Criteria

*Inclusion Criteria*. (1) Study type: randomized controlled trial; (2) study subjects: lung cancer patients; (3) interventions: radiotherapy and/or chemotherapy, supplementation of *ω*-3 PUFAs; (4) outcomes: nutritional indicators included weight change (kg), albumin change (g/L), energy intake at the end of the intervention (kcal/d) and protein intake at the end of the intervention (g/d); inflammatory indicators included CRP change (mg/L) and tumor necrosis factor-alpha (TNF-*α*) change (pg/ml). *Exclusion Criteria*. (1) Replicated published literature; (2) unable to obtain the full text of the literature with incomplete data; (3) patients not under radiotherapy and/or chemotherapy; (4) lung cancer surgery patients.

### 2.3. Data Extraction

Two researchers screened the literature, extracted data according to the inclusion and exclusion criteria mentioned above, and consulted with the third researcher in case of disagreement. The following data were extracted from the literature: first author, year, country, region, population, treatment method, experimental intervention method, intervention time, intervention dose, sample size, and clinical outcomes.

### 2.4. Risk Assessment

The included studies were assessed for risk of bias using the Cochrane Handbook (version 15.1). These included the random sequence generation method, allocation concealment, blinding of patients and trial personnel, blinding of outcome assessors, data integrity, selective reporting, and other biases. We used Revman 5.3 to generate a risk of bias graph. The GRADE method was used to assess the quality of evidence for different outcomes.

### 2.5. Statistical Analysis

Some studies showed the median and interquartile range results, and we used Wan et al.'s method to calculate the mean and standard deviation (SD) [10]. Some studies did not give the SD of the difference before and after the intervention, and we used the method recommended by the Cochrane Handbook (version 15.1) to calculate the SD. Meta-analysis was performed using Stata 15.1 statistical software, and all outcomes were calculated (SMD and 95% confidence intervals). The *Q* test and *I*^2^ statistical values were used to evaluate heterogeneity. *I*^2^ values of <25%, 25%–50%, 50%–75%, and >75% indicate no, low, medium, and high heterogeneity, respectively [11]. A *Q* test of *P* < 0.1 indicated significant heterogeneity. When *I*^2^ <50% and *P* > 0.1, the fixed-effect model was selected to pool effect size, and the random-effect model was selected in other cases. For outcomes with heterogeneity, we searched for the source of heterogeneity by eliminating literature one by one or through subgroup analysis. Egger's test and funnel plot was used to evaluate publication bias.

## 3. Results

### 3.1. Search Findings

**Figure 1:**
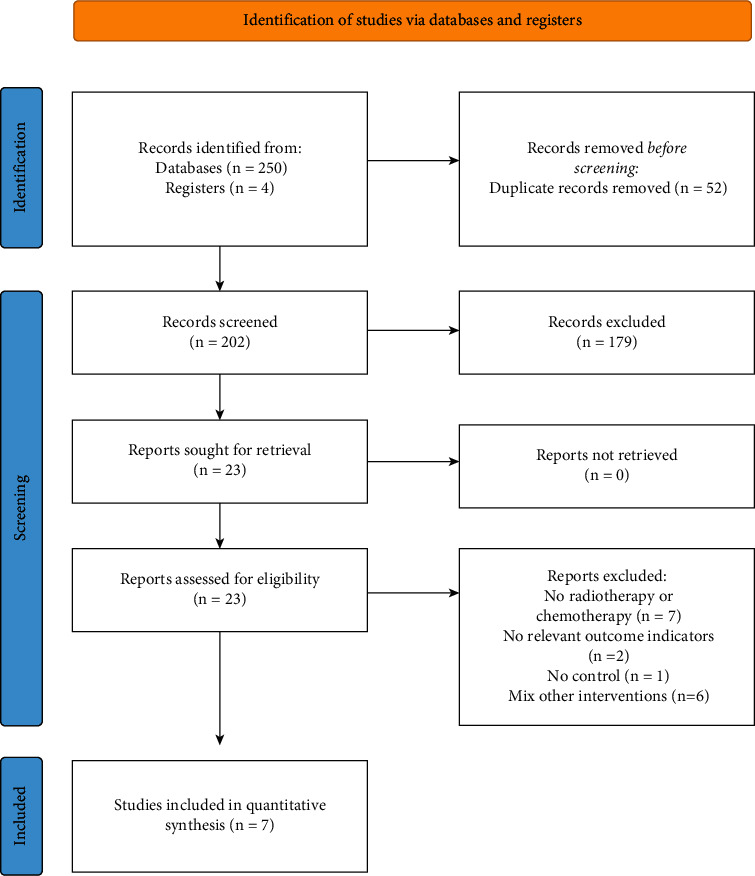
Flowchart of literature screening.

There were 254 randomized controlled trials obtained from the preliminary search. Seven studies were finally included after excluding studies not meeting the inclusion criteria. The literature screening process is shown in Figure 1.

### 3.2. Basic Characteristics of the Included Studies

Seven randomized controlled trials were included in this meta-analysis. These studies included 410 lung cancer patients, 209 in the intervention group and 201 in the control group. In the intervention groups, the intervention methods of 4 studies were EPA + DHA and 3 studies were EPA. The control groups were given a standard diet, olive oil, sunflower oil, or isocaloric nutritional supplements. The primary characteristics of the included studies are shown in Table 1.

### 3.3. Risk of Bias in Included Studies

**Figure 2:**
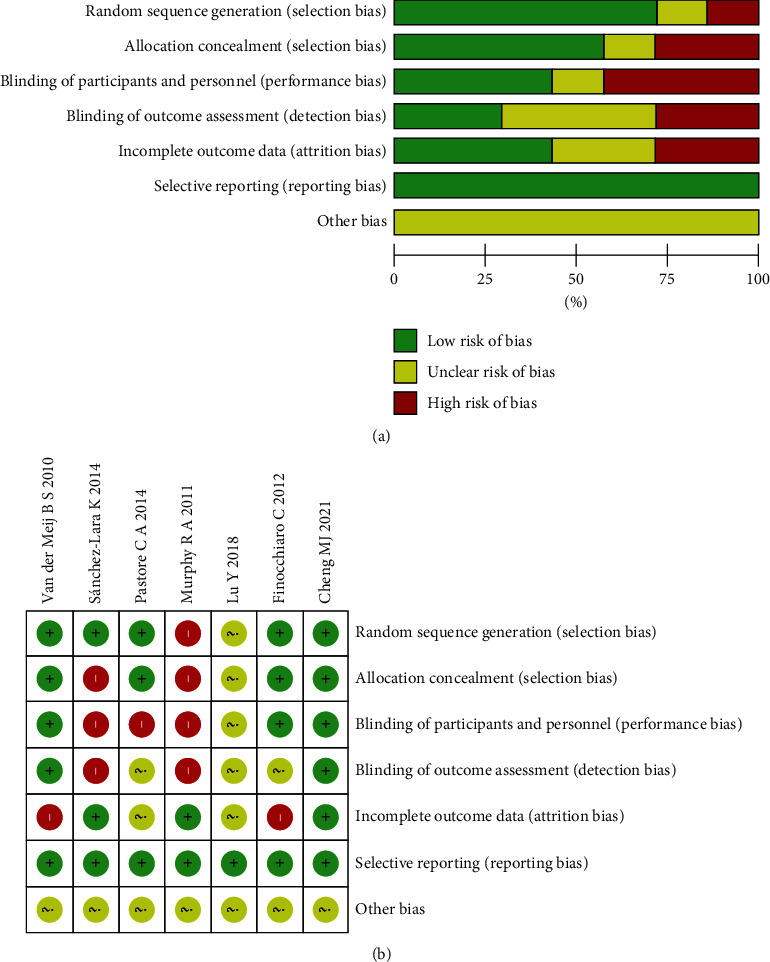
(a) Bar graph of risk of bias for included studies. (b) Overall map of risk of bias for included studies.

Of the seven included studies, 5 described the specific method of randomization, 4 described allocation concealment, 3 mentioned blinding of patients and investigators, and 2 reported lost to follow-up or withdrawal. Other sources of bias are unclear, see Figure 2 for details.

### 3.4. Meta-Analysis

#### 3.4.1. Nutrition-Related Indicators

**Figure 3:**
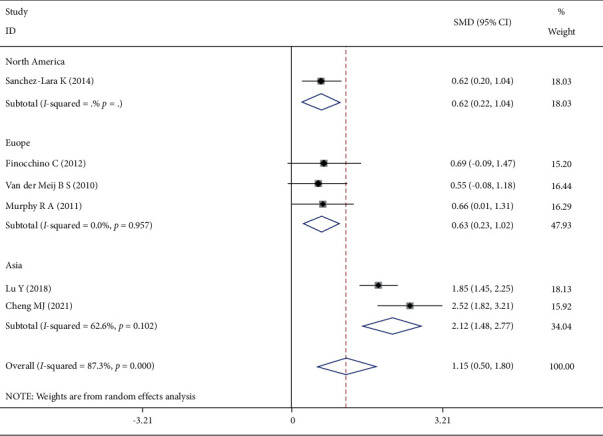
Comparison of body weight changes between the two groups of patients.

Six studies reported body weight changes, and the heterogeneity test showed that *I*^2^ = 87.3%, *P* < 0.001. The random-effect model was selected to combine the effect size, and the difference was statistically significant. Subgroup analysis by region showed that the heterogeneity among studies within the same region was small (Figure 3).

**Figure 4:**
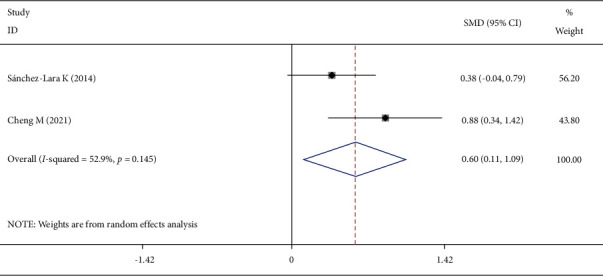
Comparison of albumin changes in the two groups of patients.

Two studies reported the albumin changes. The heterogeneity test showed that *I*^2^ = 52.9%, *P*=0.145. A random-effect model was selected to combine the effect size, and the difference was statistically significant (Figure 4).

**Figure 5:**
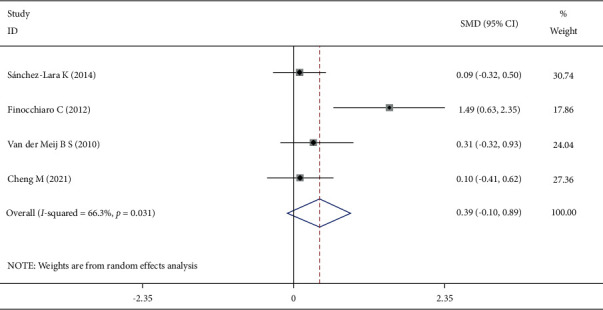
Comparison of energy intake levels in the two groups at the end of the intervention.

Four studies reported the energy intake at the end of the intervention, and the heterogeneity test showed that *I*^2^ = 66.3%, *P*=0.031. A random-effect model was selected to pool the effect size, and the difference was not statistically significant (Figure 5). Sensitivity analysis excluded the literature one by one. The heterogeneity may be derived from the study of Finocchiaro C. After the study was excluded, the SMD (95% CI) was 0.14 (−0.15, 0.42), which was still not statistically significant, indicating that the results were relatively robust.

**Figure 6:**
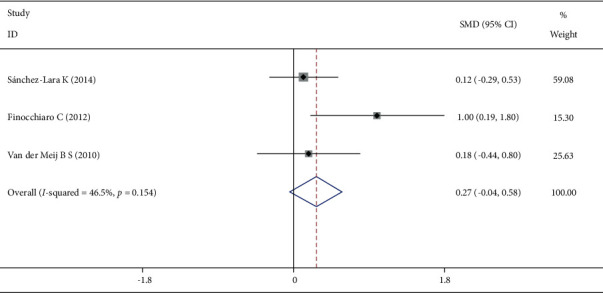
Comparison of protein intake levels in the two groups at the end of the intervention.

Three studies reported the protein intake level at the end of the intervention, and the heterogeneity test showed that *I*^2^ = 46.5%, *P*=0.154. The fixed-effect model was selected to combine the effect size, and the difference was not statistically significant (Figure 6).

#### 3.4.2. Inflammation Indicators

**Figure 7:**
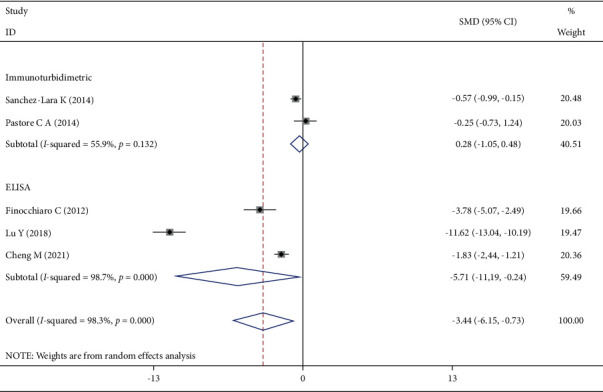
Comparison of CRP changes in the two groups of patients.

Five studies reported the CRP changes, and the heterogeneity test showed that *I*^2^ = 98.3%, *P* < 0.001. The random-effect model was selected to combine the effect size, and the difference was statistically significant (Figure 7). Studies were excluded one by one, and no obvious source of heterogeneity was found. Subgroup analysis was performed according to the detection method, and the heterogeneity of the studies with the detection method enzyme-linked immunosorbent assay (ELISA) was not significantly reduced.

**Figure 8:**
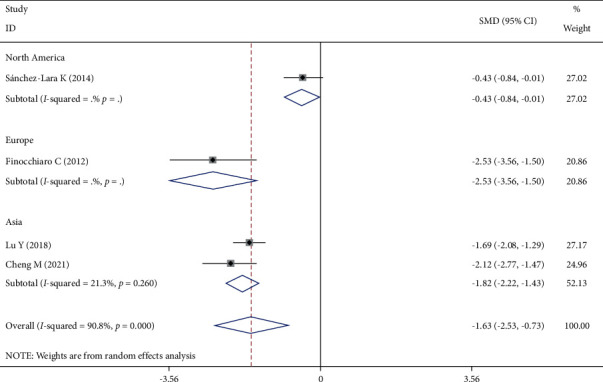
Comparison of TNF-*α* changes in the two groups of patients.

Four studies reported the TNF-*α* changes, and the heterogeneity test showed that *I*^2^ = 90.8%, *P* < 0.001. The random-effect model was selected to combine the effect size, and the difference was statistically significant. Subgroup analysis by region showed little heterogeneity among studies in the same region (Figure 8).

### 3.5. Publication Bias

When three or more studies were included for an outcome, we used Egger's test and funnel plot to assess publication bias. Egger's test showed that the *P* values were ≥0.1, indicating no publication bias. However, there are obvious asymmetries in the funnel plots of some outcomes, especially energy intake at the end of the intervention and CRP change. Egger's test results and funnel plots are shown in Supplementary Figures 1–5.

### 3.6. Evidence Quality Rating

The GRADE approach was used to assess the quality of evidence for all outcomes. The results showed that the quality of evidence for one outcome was moderate, evidence of three outcomes was low, and evidence of two outcomes was very low, as shown in Table 2.

## 4. Discussion

Patients with malignant tumors have elevated catabolism and enhanced resting energy expenditure [19]. Radiotherapy and chemotherapy can cause oral mucosal damage and dysgeusia in cancer patients, resulting in reduced food intake. Patients may experience a decrease in body weight and lean body mass and a decrease in albumin and prealbumin levels, resulting in malnutrition and even cachexia. Malnutrition is an important factor affecting the quality of life and prognosis of cancer patients. *ω*-3 PUFAs can affect the resting energy expenditure of patients by regulating the inflammatory response. They can also combine with the muscle fiber cell membrane and its intracellular organs to prevent the loss of muscle protein and upregulate the synthesis of muscle protein [20]. Studies have shown that *ω*-3 PUFAs also have a certain impact on intestinal health and its microbial composition and play a role in regulating digestion and absorption [21]. The European Society for Clinical Nutrition and Metabolism (ESPEN) recommends that cancer patients be supplemented with *ω*-3 PUFAs and fish oil to improve appetite and intake and to alleviate body weight and lean body mass loss, but the recommended level is low [22]. The meta-analysis of Ma et al. included 11 randomized controlled trials. The results showed that *ω*-3 PUFAs could significantly increase body weight and lean body mass and reduce resting energy expenditure in patients with pancreatic cancer [23]. The meta-analysis by Mocellin et al. showed that the efficacy of *ω*-3 PUFAs in increasing serum albumin was better than that of the control group [5]. However, Wan et al. study showed that *ω*-3 PUFAs did not improve nutritional indicators such as body weight, body mass index (BMI), and serum albumin compared with controls [24]. Regarding the effect of *ω*-3 PUFAs on the food intake of cancer patients, the study by Abu et al. showed that supplementation with fish oil was beneficial to the energy intake of children with leukemia [25]. In contrast, the study by Gómez-Candela et al. showed that EPA could not enhance appetite or food intake [26]. In this study, omega-3 PUFAs increased body weight and albumin levels, but energy and protein intake did not increase significantly.

There was significant heterogeneity in body weight change and energy intake at the end of the intervention. After subgroup analysis by region, the heterogeneity of weight change was reduced. However, heterogeneity still exists between the two Asian studies (*I*^2^ = 62.6%). We observed the two studies from Asia, one in patients with stage 1–4 lung cancer and the other with stage 3. This seems to suggest that region and lung cancer stage may be influential factors and a possible source of heterogeneity in weight change. Heterogeneity of energy intake was significantly reduced after sensitivity analysis excluded Finocchiaro et al.'s study. Finocchiaro et al. study has a much higher proportion of males than other studies. Therefore, we considered gender as a possible influencing factor and source of heterogeneity in energy intake.

Inflammation is considered a hallmark of cancer, and multiple studies have demonstrated that chronic inflammation is an important factor affecting tumor progression and treatment efficacy [27]. CRP is one of the inflammatory markers that cancer patients focus on, and high CRP levels may be associated with poor prognosis in lung cancer patients [28]. TNF-*α* can act as an endogenous tumor promoter and participate in promoting and developing human cancers [29]. *ω*-3 PUFAs can inhibit the activation of epidermal growth factor receptor (EGFR), thereby inhibiting the phosphorylation of growth factor receptor-binding protein 2 (Grb2), and play a role in inflammation inhibition [30]. *ω*-3 PUFAs can also inhibit inflammation by inhibiting toll-like receptors, downregulating the NF-*κ*B signaling pathway, and reducing the expression of inflammatory genes [31]. Mocellin et al. showed that *ω*-3 PUFAs reduced CRP levels in patients compared with controls [32]. Zhu et al.'s study showed that *ω*-3 PUFAs reduced TNF-*α* levels [33]. However, in a randomized controlled study by Solís-Martínez et al. that included 64 tumor patients, omega-3 PUFAs had no significant advantage in the downregulation of CRP and TNF-*α* compared to controls [34]. In our study, CRP levels and TNF-*α* levels were significantly decreased, suggesting that *ω*-3 PUFAs may have some regulatory effect on the inflammatory status in patients treated with lung cancer radiotherapy and chemotherapy.

There was significant heterogeneity in CRP change and TNF-*α* change. After subgroup analysis by region, the heterogeneity of TNF-*α* change was reduced. There were only four studies with the outcome of TNF-*α* change, and the subgroup analysis was divided into three groups. Care should be taken when interpreting the results. After subgroup analysis by the detection method, the heterogeneity of CRP change in the studies with the detection method ELISA was not significantly reduced. We observed that, of the three studies using ELISA as the detection method, one study had a higher proportion of males and the other had a different tumor stage than the others. Therefore, we considered that the detection method, gender, and tumor stage might be influential factors and a possible source of heterogeneity in CRP change.

## 5. Strength and Limitations

This study is the first to explore efficacy of *ω*-3 polyunsaturated fatty acids in patients with lung cancer undergoing radiotherapy and chemotherapy through meta-analysis. The included studies were randomized controlled trials, and the quality of the evidence was assessed. Nevertheless, there remain some limitations in our study. The implementation of blinding in included studies was unclear, and the quality of the literature was not high. Many outcomes have heterogeneity, and sensitivity analysis and subgroup analysis have found some sources of heterogeneity, but some sources of heterogeneity are still unclear. The retrieval of this study was mainly in Chinese and English, which may lead to bias. Although Egger's test does not suggest publication bias, it is still unclear whether Egger's test is suitable for evaluating publication bias when too few studies are included. In addition, the funnel plot of many results is asymmetric.

## 6. Conclusion

*ω*-3 PUFAs may improve the nutritional status and inflammatory reaction in patients undergoing radiotherapy and chemotherapy with lung cancer. The total number of studies included in this paper was small, the quality of the literature was limited, and some outcomes had large heterogeneity. Therefore, more high-quality studies are still needed to verify this conclusion and provide a reference for the adjuvant treatment of lung cancer radiotherapy and chemotherapy patients.

## Figures and Tables

**Table 1:** Characteristics of the included studies.

Author	Year	Country	Area	Diagnosis	Basic treatment	Intervention	Intervention duration	Dose	Control	Intervention sample	Control sample	Outcomes
Sánchez-Lara et al. [12]	2014	Mexico	North America	NSCLC	Chemotherapy	EPA	6 weeks	EPA 2.2 g/d	Standard recipe	46	46	abcdef

Finocchiaro et al. [13]	2012	Italy	Europe	NSCLC	Chemotherapy	EPA + DHA	66 days	EPA 510 mg + DHA 340 mg/d	Olive oil	13	14	acdef

Van der Meij et al. [14]	2010	Netherlands	Europe	NSCLC	Chemotherapy + radiotherapy	EPA + DHA	5 weeks	EPA 2.02 g + DHA 0.92 g/d	Isocaloric supplements	20	20	acd

Lu et al. [15]	2018	China	Asia	NSCLC	Chemotherapy + radiotherapy	EPA + DHA	6 weeks	EPA 510 mg + DHA 200 mg/d	Common diets	77	60	aef

Murphy et al. [16]	2011	Canada	Europe	NSCLC	Chemotherapy	EPA	6 weeks	EPA 2.2 g/d	Common diets	16	24	a

Cheng et al. [17]	2021	China	Asia	NSCLC + SCLC	Chemotherapy	EPA + DHA	12 weeks	EPA 1.6 g + DHA 0.8 g/d	Sunflower seed oil	29	29	abcef

Pastore et al. [18]	2014	Brazil	South America	NSCLC + SCLC	Chemotherapy	EPA	4 weeks	EPA2.2 g/d	Isocaloric supplements	8	8	e

Note: NSCLC: non-small-cell lung cancer; SCLC: small cell lung cancer; a: weight change; b: albumin change; c: energy intake at the end of the intervention; d: protein intake at the end of the intervention; e: CRP change; f: TNF-*α* change.

**Table 2:** GRADE quality of evidence.

Outcomes	No. of studies	Intervention sample/control sample	Certainty assessment					Certainty
			Risk of bias	Inconsistency	Indirectness	Imprecision	Other considerations	
Weight change	6	201/193	Serious	Serious	None	Not serious	None	⊕⊕〇〇 low
Albumin change	2	75/75	Serious	Not serious	None	Serious	None	⊕⊕〇〇 low
Energy intake at the end of the intervention	4	108/109	Serious	Serious	None	Serious	Serious	⊕〇〇〇 very low
Protein intake at the end of the intervention	3	79/80	Serious	Not serious	None	Not serious	None	⊕⊕⊕〇 moderate
CRP change	5	173/157	Serious	Serious	None	Serious	Serious	⊕〇〇〇 very low
TNF-*α* change	4	165/149	Serious	Serious	None	Not serious	None	⊕⊕〇〇 low

Note: evidence level downgrades one level when marked as serious.

## Data Availability

The data in the article can be obtained from the corresponding author upon reasonable request.

## Supplementary Materials

Supplementary Materials Supplementary Table 1 shows the PRISMA 2020 checklist. The search strategy can be found in Supplementary Table 2. Supplementary Table 3 shows the additional content of Table 1: Supplementary Figures 1–5 show Egger's test and funnel plot of weight change, energy intake at the end of the intervention, protein intake at the end of the intervention, CRP change, and TNF-*α* change, respectively.
